# Prospectus for the Texas Sanitarium for Consumptives

**Published:** 1903-07

**Authors:** 


### Prospectus for the Texas Sanitarium for Consumptives.

Austin, Texas, July 1, 1903.

As members of the medical profession of this State, we have upon many occasions seen the need of a first-class sanitarium, suitable for the treatment of tuberculosis, located in some ideal climate in Texas, possessing ample railroad facilities, and at a place where everything the market affords could be readily obtained, and also possessing ample diversions, such as fishing, hunting, mining, etc.

Llano, Llano county, Texas, possesses such a climate, and to us is an ideal place for the treatment of tuberculosis. Llano is the terminus of the Austin and Northwestern Railroad, and is situated one hundred miles west of Austin, Texas, and has daily train service. This little mountain city has fifteen hundred or two thousand inhabitants, nestling at the foot-hills of the mountain peaks, and has an elevation of about one thousand feet.

About the year 1892 many mines of the purest iron ore known to the world were discovered near this city, together with other rich mineral deposits. As a result, many capitalists came to Llano and invested heavily in mineral interests, resulting in what was known as a "boom," and during this time many handsome buildings were erected which would do credit to a city of one hundred thousand inhabitants. One mile from the court house of the city of Llano, upon one of the greatest elevations, and just outside of the city limits, one of these capitalists began to have erected his suburban home, choosing for its location the most ideal spot surrounding the city. After spending some eight or ten thousand dollars in the way of improvements upon this property the collapse came, and the buildings were never completed. This is the property which has been purchased by the Texas Sanitarium Company, and which is known as the "Malone Mansion" property, consisting of sixty acres of land, with many thousand dollars worth of improvements in the form of incomplete buildings. The value of the improvements, as they now stand, is shown by the affidavit made by the architect, Mr. C. H. Page, Jr., of Austin:

"This is to certify that I have made a personal investigation and a complete itemized estimate of the improvements and buildings situated on the sixty-acre tract of land known as the Malone Mansion property, situated just outside of the city limits of Llano, Llano county, Texas. This amounted to $8076.

(Signed) C. H. PAGE, JR., Architect.

Sworn to and subscribed before me this the 17th day of June, 1903.

A. R. WALLACE,

[SEAL.] Notary Public in and for Travis County."

The Texas Sanitarium was incorporated on June 20, 1903, with a capital stock of $20,000, and the purpose of the incorporators is to establish a first-class sanitarium for the treatment of tuberculosis, to be located upon the above described property, shares to be sold at $50 each, par value, full-paid and non-assessable. One-half of the stock has already been subscribed, and when three-fourths of the stock has been subscribed contracts will be let for the completion of the buildings, and the institution will be made ready for opening at the earliest moment possible. It is the purpose of the incorporators to complete these buildings as they now stand, utilizing the same for the general buildings, including dining-room, kitchen, parlor, etc., and to erect under the shade trees surrounding the same what are known as the "Munson or Holmes Sanitary Tents," the same to be used as cottages for the patients. This method of treatment is now being used very extensively in the West, and affords the best climatic results for the treatment of consumption.

The Board of Directors, who have the management of the affairs of the institution for the first year, consists of the following named physicians: Drs. J. T. Wilson, Sherman, Texas; J. W. McLaughlin, Galveston, Texas; T. J. Bennett, Ralph Steiner and M. M. Smith, Austin, Texas.

After the institution was chartered, the directors met and elected the following officers for the first year: J. T. Wilson, M. D., Sherman, Texas, President; J. W. McLaughlin, M. D., Galveston, Texas, Vice-President; M. M. Smith, M. D., Austin, Texas, Secretary-Treasurer. The Board of Directors and officers, after the first year, will be selected by the stockholders of the company. In case a sufficient amount of money is not subscribed to carry to a successful completion the intended sanitarium plan, all amounts subscribed and paid in will be returned to the subscribers.

When you consider that Texas possesses the finest climate in the world for the treatment of tubercular troubles, and that institutions erected for the treatment of this disease in other States not only give the lowest death rate, but in addition prove to be paying investments, we can readily see why a sanitarium devoted to the treatment of this trouble in Texas, after it is once well established, would certainly prove a paying investment. Any physician in Texas who has had much experience can readily recall numbers of cases who were well able and willing to pay for service in such in institution, but were denied that privilege, because no such place had ever been established in Texas, prepared to give the best service, with the latest and most advanced methods of treatment. We appeal to the members of the medical profession to aid us in this work. We do not ask their assistance in the form of a donation, but as subscribers to an enterprise which will undoubtedly return every dollar invested, with accrued interest. The stock subscribed will be full-paid when issued, and non-assessable. We shall be glad to furnish any additional information desired.

Yours fraternally,

J. T. Wilson, M. D.,
J. W. McLaughlin, M. D.,
Ralph Steiner, M. D.,
T. J. Bennett, M. D.,
M. M. Smith, M. D.

I invite the earnest attention of my readers to this enterprise and cordially and unequivocally endorse it, and endorse the eminent gentlemen who are promoting it. They are all too well known to the medical profession to need it, but I will say that such names are a guarantee of honesty and good faith and sound judgment.—Daniel, Editor.

